# Stigmatic Microscopy Enables Low-Cost, 3D, Microscale Particle Imaging Velocimetry in Rehydrating Aqueous Two-Phase Systems

**DOI:** 10.3389/fchem.2019.00311

**Published:** 2019-05-22

**Authors:** Cameron Yamanishi, C. Ryan Oliver, Taisuke Kojima, Shuichi Takayama

**Affiliations:** ^1^The Wallace H Coulter Department of Biomedical Engineering, Georgia Institute of Technology and Emory School of Medicine, Atlanta, GA, United States; ^2^Department of Biomedical Engineering, University of Michigan, Ann Arbor, MI, United States; ^3^Department of Internal Medicine, University of Michigan, Ann Arbor, MI, United States; ^4^The Parker H Petit Institute for Bioengineering and Bioscience, Georgia Institute of Technology, Atlanta, GA, United States

**Keywords:** ATPS, rehydration, convection, ELISA, particle imaging velocimetry, stigmatic microscope, micropatterning

## Abstract

This paper describes the construction of a novel stigmatic microscope and image analysis algorithm to simultaneously analyze convective mixing both inside and outside of rehydrating μL-scale aqueous two-phase system (ATPS) droplets. Stigmatic microscopy is inexpensive and advantageous because it modifies the point-spread function of fluorescent particles to enable measurement of their 3D positions from single 2D images, without needing to take slices. In one application of the technique, the convection patterns captured clarify how different ATPS formulations succeed or fail to exclude cells for patterning. Particle flow traces reveal speed and directionality of circulation, indicating temporary eddies at the outer edge of the rehydrating droplet. In another application, the speed of circulation during rehydration was analyzed for different ATPS formulations and the results used to develop a new fast ELISA procedure. While this paper focuses on ATPS rehydration, the microscope and algorithm should be applicable to a broad range of microfluidic flows where microscale 3D convection is important.

## Introduction

Aqueous two-phase systems (ATPS) can form when immiscible polymers are mixed in water. In these systems, many cells and biomolecules partition preferentially and predictably into one phase or the other (Per-Ake, 1960). These characteristics enable separation and compartmentalization of biomolecules (Hatti-Kaul, 2001; Rito-Palomares, 2004; Iqbal et al., 2016). ATPS-assisted techniques have harnessed these characteristics to address several emerging applications such as reagent delivery to cells (Tavana et al., 2009), bacterial patterning (Yaguchi et al., 2010) and sensing (Byun et al., 2013), and selective immuno-staining of cells (Frampton et al., 2015). They have been designed using dehydrated reagents, which initiate the assay upon rehydration (Frampton et al., 2014; Eiden et al., 2016). The rehydration process of ATPS droplets may dictate reagent mixing and influence the overall assay performance (Bathany et al., 2013; Lee et al., 2016). However, the dynamic behavior of the convection within rehydrating ATPS micro-droplets has been difficult to measure (Ban et al., 2016; Bansal et al., 2017). Due to this, many ATPS-assisted applications remain in the nascent development phase, lacking consensus design principles. The ability to measure internal convective flow would prove important in the process of forming these principles, thereby enabling many applications to mature. Flow tracking approaches have been explored for other applications such as particle image velocimetry (PIV), optical coherence tomography (OCT), and confocal microscopy. PIV has been utilized to deduce the trajectory of a 2d flow by tracking beads within the flow as they are scanned using a light sheet (Lai et al., 2018). Two-camera PIV further enables 3D tracking, though it is somewhat limited by the necessary multiple viewing angles. At higher resolution, μPIV has been integrated into microscopy systems, but it requires an additional prism system to enable 3D tracking (Hagsater, 2008). OCT measures the back scattering of coherent light to produce high resolution 3D images (Fujimoto et al., 2000; Lee et al., 2016). Confocal microscopy can produce high resolution 3D images, but the z-scanning method requires long image acquisition times (Moschakis et al., 2006). Spinning disk confocal has somewhat reduced that time. These techniques have all produced remarkable advances in many fields, however it would be attractive to collect 3D information about flow dynamics from a single image on a simple, low-cost laboratory setup.

To address this need we have developed a microscopy technique called stigmatic microscopy which separates the sagittal and tangential focal planes. This is achieved by introducing an astigmatism into the microscopy system. This concept was first proposed by Huang et al. who utilized it to improve reconstruction in super-resolution microscopy (Huang et al., 2008). We proposed that the optical concept could be adapted at a lower magnification and combined with three-dimensional flow tracking software to study and optimize the flow dynamics in ATPS rehydrating droplets for ATPS-based applications. Here we present the design and validation of the stigmatic microscope, then describe our insights into the flow dynamics of rehydrating ATPS droplets and finally apply this technique to illustrate flow dynamics in a cell exclusion assay (Tavana et al., 2011) and a fast ELISA system that we have previously demonstrated for multiplex readouts (Eiden et al., 2016).

## Materials and Methods

### Modeling Stigmatic Microscope Parameters

**Figure 1:**
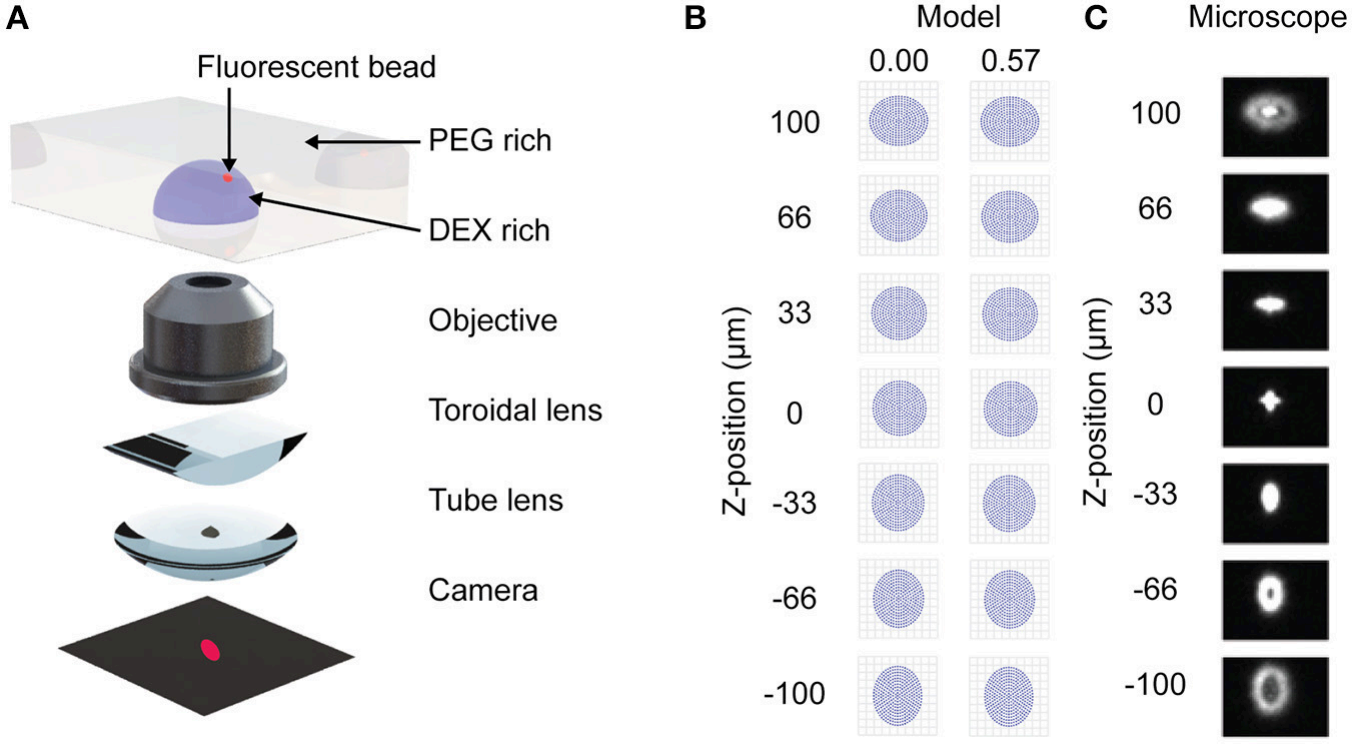
Stigmatic microscopy design and model. **(A)** Diagram showing microscope components. Adapted from Huang et al. (2008). Reprinted with permission from AAAS. **(B)** ZEMAX model of stigmatic control under microscope design for two fields (0.00, 0.57). **(C)** Confirmation of stigmatic imaging on a 3 μm bead when the bead is between −100 and 100 μm above and below the focal plane of the microscope.

As introduced before, it would be attractive to measure the convective mixing inside of a rehydrating droplet in real time. This would enable the indirect measurement of the forces in the droplet that drive convective rehydration. These forces cannot currently be measured. Here we introduce stigmatic microscopy, a microscopy technique in which astigmatic aberrations in the imaging system are used to determine the vertical position of an object relative to the focal plane (Figure 1A). When combined with bead tracking in the x and y directions we can track a bead in three-dimensions as it is carried by flow inside a droplet.

Astigmatism occurs when an oblique bundle of rays impinges a lens that does not have a symmetrical front (Seward, 2010). The resulting image appears foreshortened in the plane of incidence (tangential plane) and elongated in the sagittal plane. The difference between the image points in the tangential and sagittal planes is the astigmatic difference or the aberration called astigmatism. If the astigmatic difference is large, then the image of a bead will appear elongated in the sagittal plane above the focal plane and elongated in the tangential plane below the focal plane. Here we demonstrate that by designing an infinity corrected microscope with a prescribed astigmatism, we can derive a relationship between the position of the bead in the z direction relative to the x-y focal plane and the image of the bead.

Most modern microscopes are designed using an infinity corrected design (Seward, 2010). This term implies that the objective projects the bundle of rays it has imaged as parallel rays. In an ideal system, these would travel to infinity if not for the tube lens, which focuses these rays onto an eye piece or camera (CCD, CMOS or other). To convert an infinity corrected design into a stigmatic microscope, a toroidal lens is inserted between the objective and the tube lens (Figure 1A). Because a toroidal lens has different focal lengths in the sagittal and tangential directions it will introduce an astigmatism into the final image.

As shown in Figure S1A the paraxial layout establishes the focal lengths and overall size of the microscope. The lens system is composed of two lens groups, the objective (Nikon 4X CFI Achromat, NA 0.1) and tube lens (Thorlabs, LJ1516RM), with a stop in between them and an entrance pupil (20 mm) controlled by the objective. There is also a doublet relay lens (Thorlabs, AC254-050-A) to alter the magnification of the image onto the camera. Here *f*_1_ is the focal length of the objective, *f*_2_ is the distance between the tube lens and the relay lens, *f*_3_ is the distance between the relay lens and the camera, and *d* is the distance between the objective and the tube lens. They are arranged such that the objective is one focal length (50 mm) away from the substrate and the camera is 86.7 mm away from the tube lens. Without the relay lens this distance would be one tube lens focal length (154 mm). To introduce astigmatism, a half round toroidal lens was inserted into the system 3 mm in front of the tube lens and the system was re-focused using the RMS centroid spot size (49.7 mm). These elements were used as the basis of our idealized model of the optical system.

The paraxial model of the microscope was developed in the optical design software ZEMAX (Geary, 2002). It is important to note that some prescriptions of optical components used in experiments are proprietarily held by Nikon and data on them is unavailable, so the model presented here is for an idealized infinity corrected microscope. As an example of the contribution to astigmatism by the toroidal lens, Figure 1B shows the shape of a bead above and below the focal plane when a 1,000 mm toroidal lens is used. The strength of the toroidal lens must be carefully selected to produce a sufficient astigmatism without excessively disrupting image quality, as expressed by the modulation transfer function (Figure S1C). We demonstrate how the strength of the toroidal lens can be mapped to the distance between the best focus for the sagittal and tangential directions (Figures S1D,E). This data is necessary to calibrate the microbead z-position from the image (Figure 1C).

### Constructing Stigmatic Microscope

For our test microscope we adapted an inverted Nikon TS100 microscope by inserting off the shelf components from Thorlabs (TTL, a 200 mm tube lens and LJ1703RM, a 1,000 mm toroidal lens) as guided by the model. The focal length of the tube lens was chosen to match the Nikon 200 mm focal lengths specified by the company. The microscope was constructed by unthreading the Nikon tube lens and replacing it with the Thorlabs toroidal and tube lenses, respectively. The toroidal lens was placed 3 mm in front of the tube lens. Note that the rotational alignment of the toroidal lens with the camera is critical for subsequent analysis.

### Calibrating Microbead z Position

**Figure 2:**
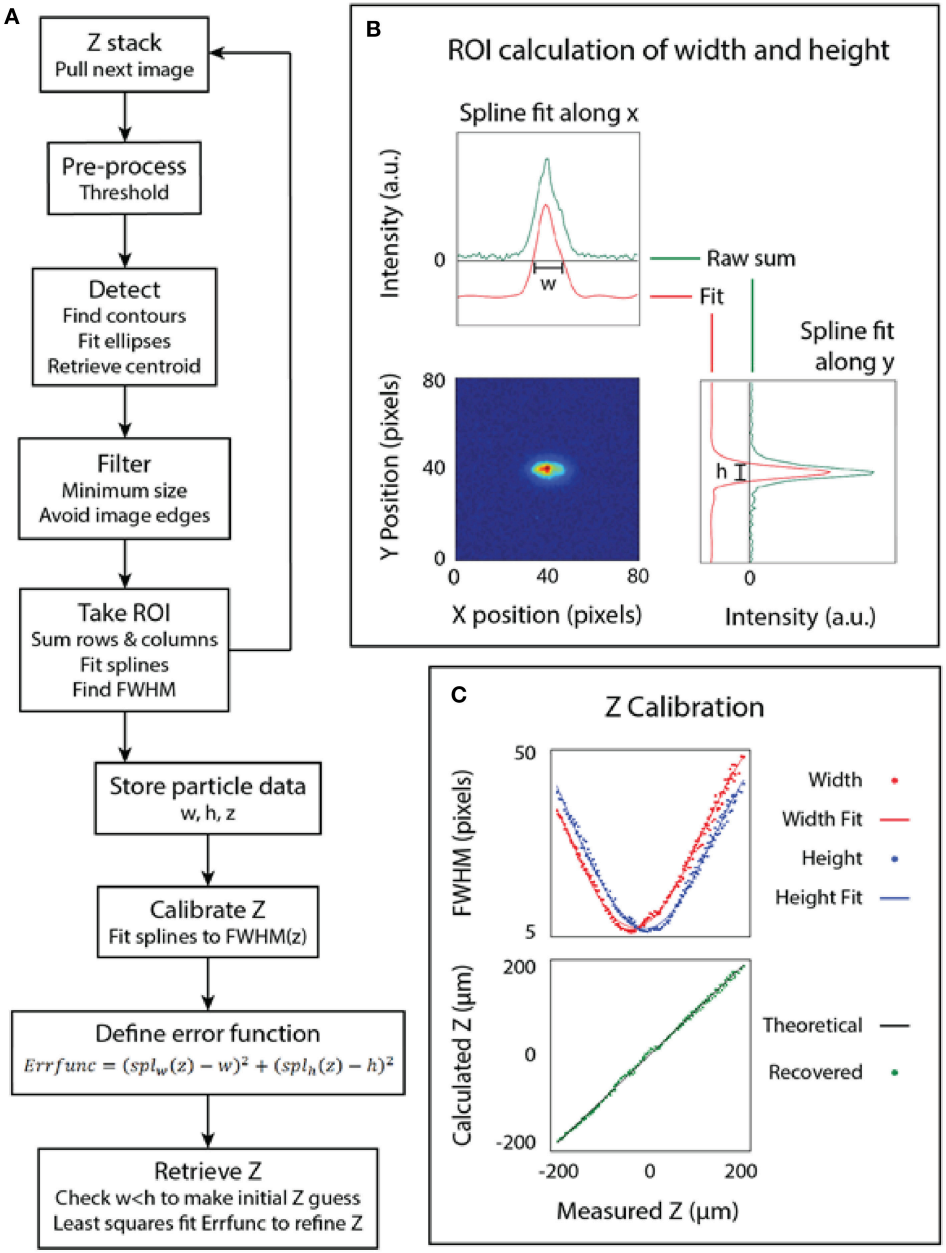
Calibration of the microscope. **(A)** The algorithm used to calibrate the microscope. **(B)** The point spread function (measured and fit) of a bead. **(C)** The bead dimensions plotted against the focus position (z) and the final calibrated relationship.

To calibrate the microbead z position from a captured image we measured the astigmatic aberration using an algorithm in Python and the library OpenCV (Figure 2A) (Bradski and Kaehler, 2008). A single 3 μm yellow-green fluorescent bead was placed in a 96-well plastic microplate inside a droplet and allowed to settle to the bottom of the well (Figure 2B). Images were taken at 5.5 μm intervals from −200 μm below the bead to 200 μm above the bead, then fed into the software algorithm.

The z-calibration loop collected particle height and width for each image, then appended the particle dimensions to an array labeled by z-stack position, as follows. For each image, a threshold was used to generate a binary image. From the binary image, a bounding ellipse was fit to the bead dimensions to find the center of the bead using the built-in functions in OpenCV (Fornaciari and Prati, 2012). The particle was found using blob detection and a region of interest (ROI) was used to extract just the particle from the image, as depicted in Figure 2B. A line histogram was applied along the x and y axes of the image to project the 2D point spread function of the bead. A smoothing spline was fit to calculate the full width at half maximum (FWHM) of the data. The line histogram data was filtered to only include lines two sigma from the mean to filter noise. The distance between the roots of the spline provided the width and height of the bead. This algorithm enables simultaneous detection of multiple beads at multiple z heights from the same image, but overlapping beads can interfere with each other.

This same technique was applied after imaging at known intervals above and below the focal plane to produce a scatter plot of the width and height of the bead at different z positions. The result of fitting a spline to this data is the calibrated relationship of the PSF of the bead to its position relative to the focal plane of the objective. The z position of an unknown bead can then be calculated using a least-squares fit on the calibrated relationship, which attempts to minimize the error between both the width and height of the bead to the calibrated model. Note that the least-squares fit on the spline function is somewhat sensitive to initial z estimate, so we pre-filtered the z estimate by fitted ellipse area and by width to height ratio. Finally, we plot the measured z-position of several beads to the known z-position of those beads that were back calculated, as shown in Figure 2C.

### Preparation of DEX–PEG Aqueous Two-Phase Systems

Two aqueous two-phase systems were selected, based on prior experience in ATPS micropatterning. Our group has previous published work on the multiplex ELISA and cell exclusion assay using PEG (MW 35,000, Sigma) and DEX (MW 500,000, Sigma) (Tavana et al., 2011; Frampton et al., 2014). Flows in a PEG and Ficoll (MW 400,000, Sigma) system were also examined. We generated phase diagrams for both systems using a conventional dilution method (Kojima and Takayama, 2013). Briefly, we prepared concentrated polymer solutions in PBS (20% w/w polymer) and mixed the biphasic solutions in a range of different volume ratios. The resulting cloudy mixtures (two-phase) were diluted by a PBS solution until the binodal point where the mixtures turned transparent (one-phase). A univariate spline was fit to the resulting set of points to indicate the binodal curve (Kojima and Takayama, 2013). Separate solutions above the tie-line were prepared to measure the resulting top phase/bottom phase volume ratio. Using the conservation of mass, the volume ratio can be calculated from the ratio of distances between the bulk concentration and either intersection of the tie-line with the binodal curve. Tie-lines were fit to the phase diagram using a custom Python script. A least-squares fit was used to find the slope of the tie-line that matched the volume ratio calculated by the relative tie-line lengths to the measured volume ratio. The intersection of the tie-line with the binodal curve was found by least-squares fitting between the binodal spline and the tie-line with a fitted slope that passed through the bulk concentration point. Note that the univariate spline does not accurately describe the binodal curve at near-zero values of the independent variable, so an inverse spline was also found and used for the opposite end of the binodal curve, i.e., y(x) for low values of y and x(y) for low values of x. Phase diagrams are included in Figure S2.

We measured convection in rehydrating ATPS that mimics the situation seen in the multiplex immunoassay format (Frampton et al., 2014; Eiden et al., 2016) and cell exclusion patterning (Tavana et al., 2011). Solutions of the DEX phase in DI-water were spotted in 1 μL droplets in a clear, flat-bottom 96-well microplate with 3 μm yellow-green fluorescent polystyrene beads (polysciences), diluted to ~10–20 particles per μL. The plates were dried in a vacuum desiccator overnight prior to rehydration. DEX microdroplets were rehydrated on the stigmatic microscope with indicated concentrations of PEG in PBS and imaged over indicated times. For flow exclusion studies, beads were diluted to 10–20 particles per μL in the PEG-rich phase to track flow outside of the rehydrating droplet.

### Flow Tracking

**Figure 3:**
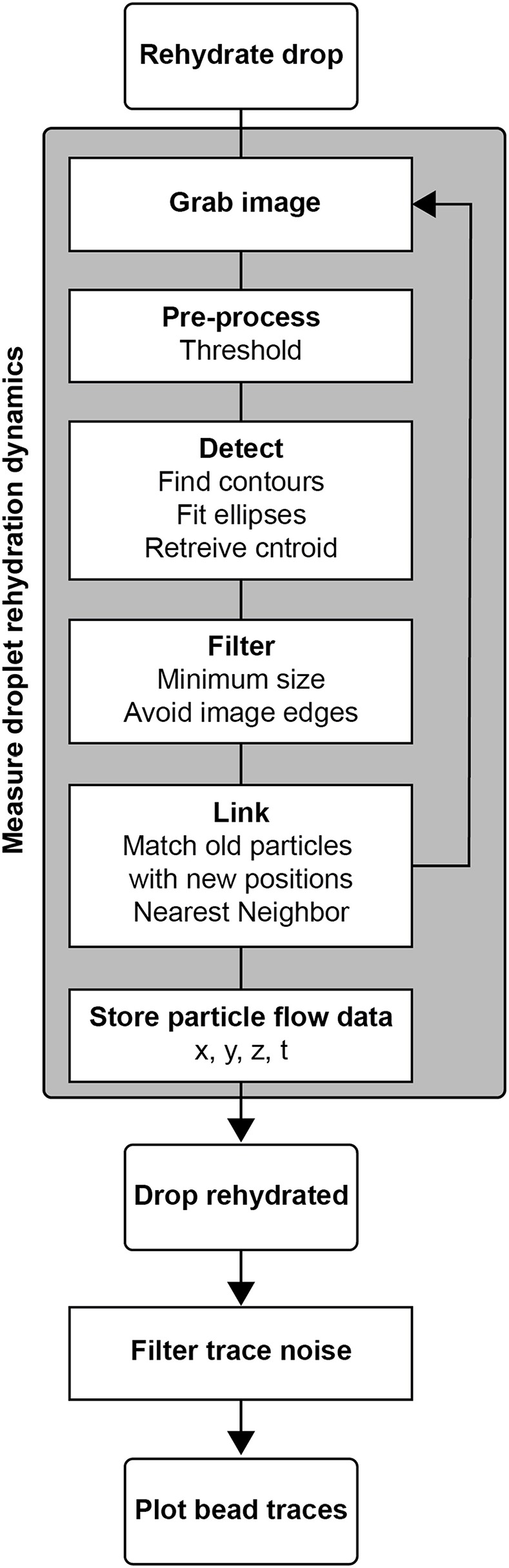
Algorithm for measuring droplet rehydration dynamics in stigmatic microscopy. The process for measurement involved rehydrating the drop under microscopic imaging, during which the particles were tracked, and their X, Y, and Z positions were reported.

Time course images were collected using Nikon NIS Elements and saved as 16-bit .nd2 files. These files were converted to 8-bit .tiff images, conserving the low intensity region of the data using a FIJI macro. We wrote analysis in Python to calculate 3D bead positions and track their movement (Figure 3).

The image thresholding and bead position calculation followed the same algorithm as in the calibration protocol. For each period t, several bead positions were calculated and indexed. Each existing bead was then compared to beads in frame t+1 and linked to the nearest neighbor by x, y, z distance in microns, checking against a maximum travel distance threshold. If a match was found, that bead pair was removed from subsequent matching to prevent flow traces from artificially merging. The remaining beads from period t were similarly matched to the remaining beads in frame t+1. Any beads that were not matched were held for five time frames before terminating that bead path. The resulting flow trace matrix contained x, y, z positions, time points, and bead identity indices. Traces containing fewer than five hundred time points were eliminated as noise.

For flow exclusion studies, the field of view was aligned to contain the outer edge of the droplet. To view the flow at the droplet interface, a 400 px (600 μm) slice was taken by filtering the trace data in the y-dimension. The traces were then projected to the x-z plane. Flow traces were color-coded by time to visualize the direction of flow.

Flow traces were imported to MATLAB and smoothed using a Loess filter in x, y, and z. Once smoothed, the time derivative of 3D distance traveled could be calculated numerically to find particle speed. For the 15-min traces, the root-mean-square of the speed was calculated for each particle to find a general metric for mixing efficiency. To distinguish between the fast mixing eddy regions and the larger central flow, flow traces were plotted in a 3D interactive diagram using open-source plotly functions, color-coded by time, and examined for annotation. To determine droplet rehydration height, traces were examined in the interactive plots, as well as using x-z projections.

### Cell Exclusion

**Figure 5:**
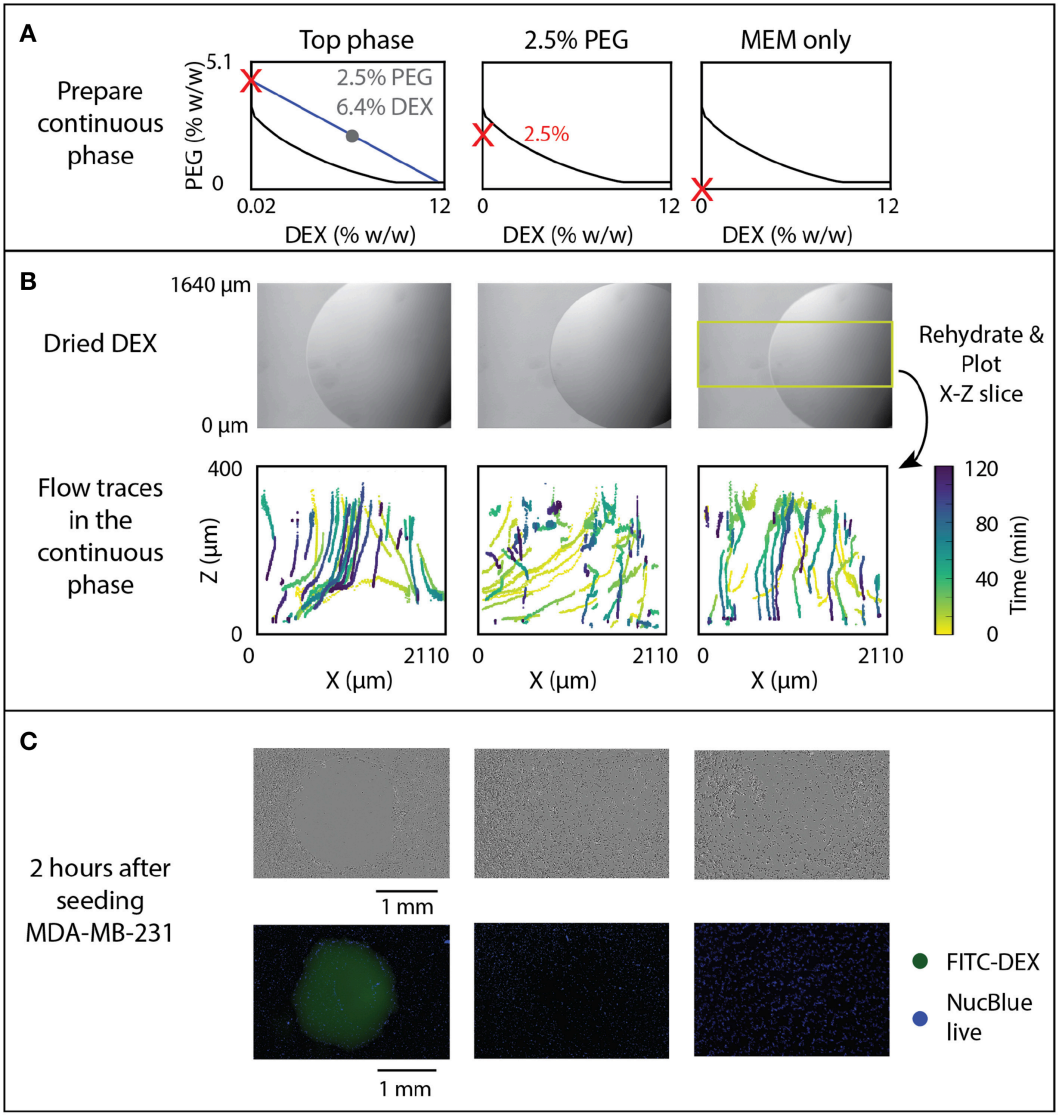
Effect of rehydration dynamics on cell exclusion. **(A)** ATPS solutions indicated by red Xs were used to rehydrate DEX droplets, shown prior to rehydration in panel **(B)**. **(B)** Flow traces of beads in the continuous phase, with color-coded time, were measured and 3D sections indicated by the yellow box were projected to the X-Z plane. **(C)** Cell exclusion was imaged 2 h after seeding MDA-MB-231 cancer cells using phase contrast (top) and fluorescent imaging with FITC-labeled DEX and NucBlue Live Stain (bottom).

Cell exclusion assays were performed by rehydrating 1 μL DEX drops in which the PEG phase also contained MDA-MB-231 cells (ATCC) to observe if the DEX spotting would exclude the cells from adhering to the spotted region. Three conditions were studied, including a 2.5% PEG-6.4% DEX (w/w) ATPS system, a 2.5% (w/w) PEG only system, and MEM-only system. The DEX was prepared first at 6.4% (w/w) in DMEM F12 (Invitrogen) labeled with FITC-DEX (0.1% w/w, Sigma). Each solution was filtered (0.22 μm PVDF syringe filter) in a sterile environment after the reagents had been prepared and dissolved. The 2.5% PEG-6.4% DEX system was pre-equilibrated by centrifugation at 25°C, 3,000 rpm for 15 min, and the top phase was extracted for cell exclusion experiments. The resulting cell suspension solutions are indicated in Figure 5A.

The DEX solution was spotted in 1 μL drops into clear, flat-bottom 96-well tissue culture microplates using an Integra Biosciences Viaflo 96-well pipetting robot (pipet function, speed 8 with a Z height of 11.2 for the receiving plate) and were dried overnight in the incubator to maintain sterility. The top phase of the 2.5% PEG-6.4% DEX solution or the 2.5% PEG only or MEM-only solutions were prepared in DMEM F12 as described above. Cells were resuspended in these solutions at a concentration of 400,000 cells/mL, then added to the dried DEX spot plate to rehydrate the DEX. The cells were stained with NucBlue Live (Thermofisher) according to the manufacturers protocol. The plates were incubated for 2 h at 37°C to allow cell settling, then imaged.

## Atps Elisa

The current work adapts the protocol from our group's previous publication (Eiden et al., 2016) using a human IL-8 DuoSet kit (R&D Systems). ELISAs were performed in custom 96-well injection molded plates with 1.7 mm diameter microbasins (9 per well) (Xcentric Mold and Engineering). Plates were washed with ethanol and distilled water, then dried overnight in a desiccator. Capture antibody solutions at the manufacturer recommended concentration in PBS (4 μg/mL) were applied in 1 μL spots to the microbasins. The plates were sealed and incubated for 90 min to allow adsorption. Free capture antibody was then washed 3x using 200 μL wash buffer (0.05% Tween 20 in PBS), followed by addition of 100 μL of 5% sucrose (Sigma) in PBS for 1 h to block unused surfaces. The sucrose was emptied, and the plates were dried in a vacuum desiccator for 40 min. Once dry, 5% BSA in PBS was spotted 1 μL per microbasin on top of the IL-8 capture antibody spots to further block reaction surfaces. The BSA was dried in the vacuum desiccator for 40 min. Detection antibody was mixed in solutions of 5% (w/w) dextran MW 500,000 in water (Sigma). These detection antibody solutions were then spotted 1 μL per microbasin and dried in a vacuum desiccator overnight.

To run the assay, IL-8 standards from the kit were diluted in an assay buffer consisting of 5% (w/w) or 10% (w/w) PEG MW 35,000 (Sigma), 0.05% Tween 20 (Sigma), and 0.5% BSA (Sigma) in PBS (Gibco). An 8-point standard curve was generated with 2-fold dilutions from 2,000 pg/mL in quadruplicate. To avoid biasing the plate by sample addition order in the fast ELISA, samples were loaded onto a feeder plate in a randomized plate layout. The samples were then simultaneously transferred from the feeder plate to the assay plate using the Viaflo 96-well robotic pipet. The assay was initiated upon addition of the PEG-sample solutions, which immediately began to rehydrate the dried DEX and antibody reagents. Samples and standards were incubated in the dark at room temperature for 15 min. The plates were washed 6x with wash buffer to clear out the viscous ATPS components. Streptavidin-HRP conjugate (R&D Systems) was added at the manufacturer's recommended concentration and incubated for 20 min. Plates were washed 3x, then filled with SuperSignal ELISA Femto Maximum Sensitivity Substrate (ThermoFisher) and the plates were imaged using a BioRad ChemiDoc MP+ chemiluminescent blot reader.

ELISA plate images were analyzed using a custom ImageJ plugin to outline microbasin regions and calculate chemiluminescent intensity. The standard curve was plotted using the ggplot2 library in CRAN-R. ANOVA was used to test effects of row, column, ATPS condition, and IL-8 concentration in a randomized plate design with a confidence interval of 95%. Following ANOVA, a *post hoc* Tukey test was performed with a 95% confidence interval. A linear model was used to fit the data and remove effects from row and column location within the plate to plot the standard curve.

## Results And Discussion

### Stigmatic Microscope Validation

Z-stacks from an individual fluorescent bead indicated that the stigmatic microscope transformed the point spread function (PSF) as expected (Figure 2B; Figure S1B). Figure 1B shows sample images taken along a stack of z positions, where the spread of light follows the shape predicted in the spot diagram. The 3 μm bead diameter corresponds to 2 pixels in image-space, making it a reasonable approximation for a PSF. Smaller beads were also examined, but the tradeoff between fluorescent intensity and size led to the selection of the 3 μm bead. The 3 μm beads are expected to respond to gravitational force for sedimentation (Laidlaw I. M. A. R. C. S., 2005) and other forces for convection (Binks and Horozov, 2006). Generally, smaller beads will be less sensitive to gravitational effects and therefore better for flow tracking. However, small beads contain less dye and have low fluorescent intensity, requiring longer exposure times to capture the shape of the PSF. Exposure can be further tuned to sacrifice depth of field for imaging frequency. For applications examining shallower flows, smaller beads may still be appropriate. In this work, a 500 ms exposure provided sufficient detail about the shape of the PSF to detect z-position within at least the calibrated 400 μm range. Faster flows requiring shorter exposure can be detected by decreasing the exposure time and sacrificing z-range or by introducing a photo-multiplier tube into the system.

After performing the calibration outlined in Figures 2A,B on 3 different beads, the images were fed back into the z-retrieval algorithm to recover z-position. Figure 2C indicates accurate recovery of the z-position.

### Rehydration Dynamics of Rehydrating ATPS Droplets

**Figure 4:**
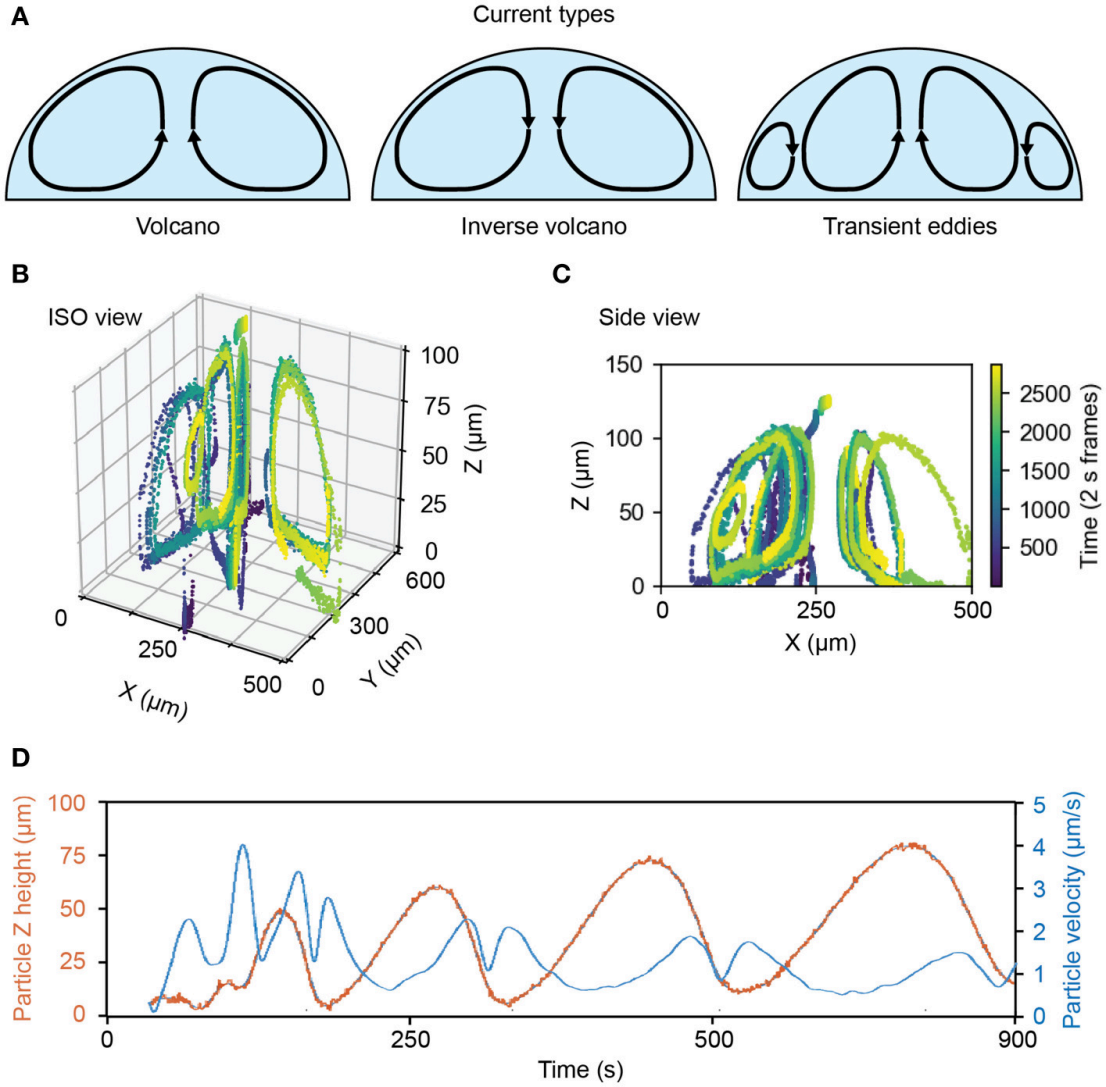
Flow dynamics and rates of rehydrating ATPS droplets. **(A)** Examples of observed flows inside of rehydrating ATPS droplets. **(B)** 3D plot showing the flow dynamics of an exemplary rehydrating Ficoll-rich droplet. **(C)** 2D slice of the center of the same Ficoll drop over several flow cycles. **(D)** Dynamics and rate of rehydration as measured for a drop showing particle positions over time (red) and particle velocity over time (blue) in the drop.

Three primary types of flow dynamics emerged during the rehydration process (Figure 4A). The first was the volcano current, in which direction of flow is up the center of the droplet. The second is the inverse volcano, in which the current flows down the center of the droplet. We also observed transient micro eddies in high mixing regions where particles would rapidly rotate near the bottom, outer edge of a droplet.

Inverse volcano currents were observed in PEG-Ficoll ATPS drops while both volcano and inverse volcano currents were observed in PEG-DEX ATPS drops, depending on specific ATPS composition. Interestingly, the micro eddies were only present in the DEX ATPS drops. Figures 4B,C show an example of a PEG-Ficoll inverse volcano. Figure 4B shows the ISO view of particles being tracked in the droplet over 50 min. Figure 4C shows the side view of the same droplet in which individual beads show a uniform current around the drop. This is just an example of the measurements that are observed using the stigmatic microscope system.

For many applications, knowing the velocity of the current and the cycling rate of the fluid in the drop may be useful for optimizing conditions. Figure 4D shows an example of the flow dynamics of a bead in the fluid. The z position of the bead in this example increases in both amplitude and period with time. Moreover, the velocity of the bead shows a double peak pattern after reaching the peak of the drop. The maximum z-position the particle reaches increases with time in this case from 50 to 75 μm, and it is inversely proportional to the maximum velocity per flow cycle, as the droplet becomes more well-mixed and loses some of the chemical potential gradient that may be driving flow.

### Cell Exclusion

The correlation between flow behavior and rehydrated ATPS cell exclusion patterning was examined. Previously reported experiments indicated a change in cell exclusion efficiency depending on the composition of the ATPS. In this assay, cells in solution sediment and attach to the growth plate in a pattern excluded by a dried 1 μL 6.4% DEX spot. To further examine this behavior, we tested three continuous phase conditions, all with a dried 1 μL DEX spot in each well (Figure 5A): “Top phase”–the extracted top phase from the 2.5% PEG-6.4% DEX system, “2.5% PEG”–a sub-critical ATPS, made of 2.5% PEG, and “MEM only”–cell culture media alone. These conditions are marked by red Xs on phase diagrams in Figure 5A. Note that the sub-critical ATPS contains a small overall concentration of DEX from the dried 1 μL DEX spot. However, that overall concentration of DEX (0.064%) does not bring the total system above the binodal curve. In contrast, the “Top phase” condition yields a higher concentration of PEG, as it has already phase separated from the initial composition, indicated by following the blue line from the gray dot to the red X in Figure 5A. This higher PEG concentration brings the overall system above the binodal curve, producing equilibrium phase separation. For imaging, the field of view was shifted to include the edge of the droplet to see flow behavior at that region of interest during rehydration, as indicated in Figure 5B. In Figure 5B, the time of each detected bead position is encoded by color.

Visual examination of the flow both inside and outside of the droplet added new explanation to the success of cell exclusion patterning. In the two-phase condition (labeled “Top phase” in Figure 5), flow tracking within and around the rehydrating droplet indicates a volcano-pattern circulation within the droplet, and a downward/outward flow in the surrounding media (Figure 5B, left). This generates a small stagnant zone at the center of the droplet, which corresponds to the typical location of cells that evade exclusion and attach near the center of the droplet (Tavana et al., 2011). This experiment indicates that convective flow is present and may help drive cell seeding away from the droplet, in addition to the previously hypothesized interfacial exclusion (Tavana et al., 2011).

In sub-critical ATPS (Figure 5B, “2.5% PEG”), initial flow is similar to the “Top phase” condition, but at long times the “2.5% PEG” condition resembles the “MEM only” condition, after the ATPS dissipates and becomes homogenous. There is a transient flow exclusion for ~1 h, while the DEX-rich phase is at a temporary, locally high concentration. However, the bulk phase and DEX-rich phase will ultimately become homogeneous, as the total composition of the system is below the binodal curve. Over time, the DEX may be diffusing across the droplet interface into the bulk phase while the droplet phase is also diluted by infiltrating osmosis, reducing the local concentration of DEX below the binodal curve. Time-lapse imaging of FITC-DEX droplets indicate that the distinct droplet phase disappears within 2 h (Figure 5C). Temporal analysis of the convective flow paths shows that after ~1 h, beads settle through the area previously obstructed by the temporary phase boundary. In the absence of PEG, flow consists only of settling straight downward (Figure 5B, right).

The efficacy of cell exclusion correlates with the flow profiles (Figure 5C). Representative brightfield and fluorescence images indicate near total cell exclusion in the two-phase condition, but no noticeable exclusion in the sub-critical ATPS or the media-only control. The lack of cell exclusion in the sub-critical ATPS result indicates either that convection alone was insufficient to direct cell patterning, or that cell settling continued to occur beyond the 1 h of transient exclusion flow.

The difference between the pre-equilibrated two-phase condition and the sub-critical ATPS emphasizes the need for clarity in ATPS research when communicating ATPS concentrations, particularly near the critical point. Some papers describe the overall concentration (mid-tie-line, as in this experiment), while others describe the resulting phases used along the binodal curve.

### ATPS ELISA

The impact of ATPS composition on ELISA performance could arise from a variety of factors. These include partition behavior, volume ratio, convective mixing, and viscous limitations on diffusion. The partition coefficient and volume ratio determine the concentrating effect from ATPS (Mashayekhi et al., 2012). Partition coefficients and volume ratios further from unity cause higher concentrations of reagents into one phase, in this case the DEX phase. Techniques already exist to determine partition coefficients, volume ratios, and viscosity, but internal convective mixing has been difficult to measure.

The stigmatic microscope presented here can add missing information regarding convective mixing and transient volume ratio in non-equilibrium ATPS. We examine two ATPS conditions, with low or high PEG concentrations (5 or 10% w/w, respectively) to rehydrate a dried 1 μL droplet of 5% DEX, for overall ATPS consisting of 5% PEG and 0.05% DEX or 10% PEG and 0.05% DEX. We will refer to these conditions as 5% PEG-5% DEX and 10% PEG-5% DEX, respectively. Recent work suggests that convection in non-equilibrium ATPS is driven by a chemical potential gradient (Ban et al., 2016). The chemical potential gradient is higher for the higher concentration PEG, which lies on a longer tie-line. However, the resulting concentrations of PEG and DEX in the top and bottom phases are also higher, leading to higher viscosity and associated drag on convective flow.

**Figure 6:**
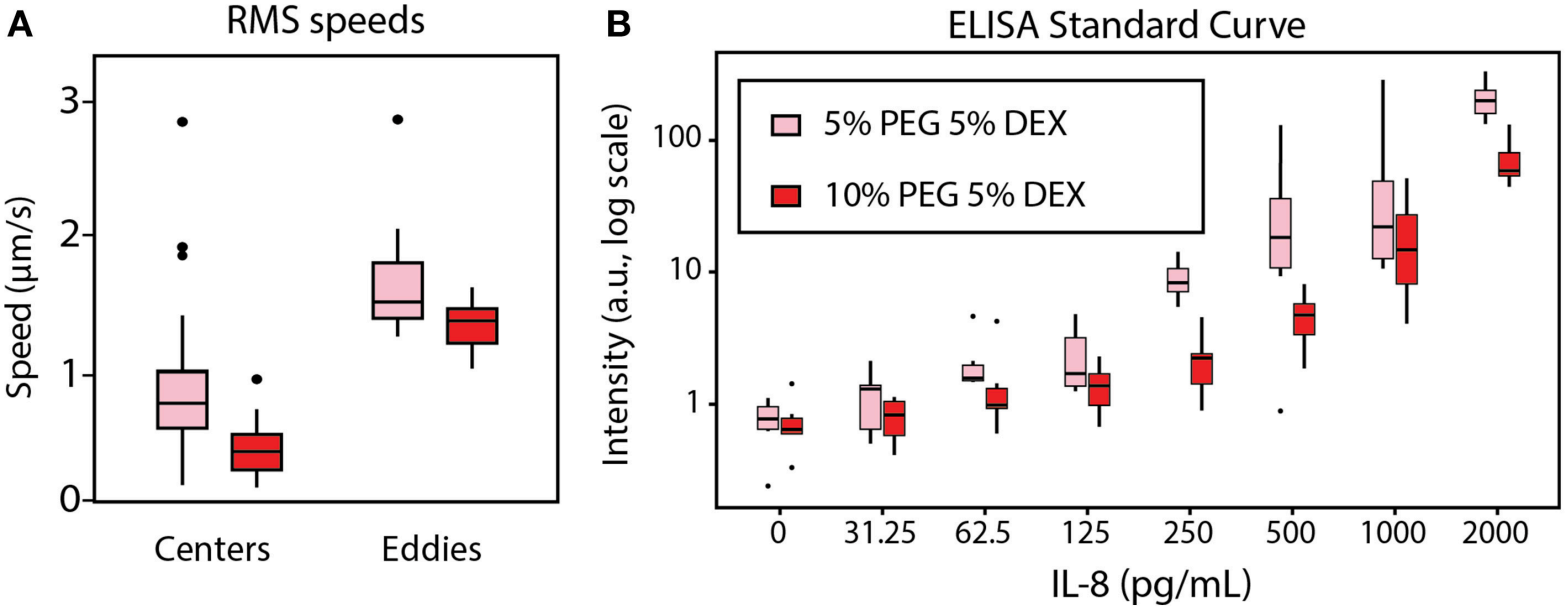
Effect of rehydration dynamics on ELISA performance between 5% PEG−5% DEX (pink) and 10% PEG−5% DEX (red). **(A)** Individual speed traces were summarized using their root-mean-square (RMS) values, then plotted in boxplots. Each eddy was significantly different from each center with a 95% confidence interval. The difference between center RMS speed was also significant at a 95% confidence interval. **(B)** The standard curve for the row-column-corrected IL-8 ELISA was determined. The difference in signal intensity between 5% PEG-5% DEX and 10% PEG-5% DEX was statistically significant at 95% confidence interval by ANOVA with a *post hoc* Tukey test.

We quantified spontaneous convective mixing in rehydrating ATPSs. As depicted in Figure 4D, flow traces were smoothed in x-, y-, and z- dimensions as functions of time for numerical differentiation to find the speed over time. Speed was then quantified using the root-mean-square (RMS) to summarize each flow trace into a single measurement. The flow traces were visually examined to annotate each trace as a short eddy or a larger, center flow. The RMS speeds were analyzed between conditions using ANOVA, followed by a *post hoc* Tukey test at a 95% confidence interval. The center flow was significantly faster for the 5% PEG-5% DEX compared to the 10% PEG-5% DEX, while the difference between eddies did not meet the 95% confidence interval (Figure 6A). We suggest that the additional drag effects from viscosity in the 10% PEG-5% DEX condition seem to outweigh its higher chemical potential gradient driving forces.

Overall droplet height was roughly identical for the 5% PEG and 10% PEG conditions (Figure S3). The indistinguishable droplet heights do indicate that potential differences in volume ratio between ATPS conditions are not present. Therefore, differences in ELISA signal may be attributed primarily to differences in convective mixing.

The extensive mixing effect seen in the 5% PEG condition correlated with higher signal intensity in the standard curve of a rehydrated ATPS ELISA for human IL-8. ANOVA with *post hoc* Tukey test indicated that the 5% PEG-5% DEX condition gave higher intensity than 10% PEG-5% DEX at a 95% confidence interval. The row-column corrected standard curve is shown in Figure 6B. We suggest that this result occurs because greater internal convection provides more binding opportunities between the detection antibodies confined within the DEX droplet and the IL-8 sample in the bulk PEG-rich phase. The convection also allows additional binding opportunities for the IL-8 sample to the capture antibody on the microplate surface.

Furthermore, we have shown an ELISA with only a 15 min incubation, 16x faster than our previously reported protocol (Eiden et al., 2016). This study demonstrates the ability to measure convective mixing using stigmatic microscopy, which will be critical for further analysis and optimization of this short incubation ELISA. These studies will include analysis of other ATPSs and their impact on signal intensity and variation.

## Conclusion

The design of the microscope is both simple and flexible, enabling it to be integrated into most off the shelf microscope systems without a deep knowledge of optics. The principle is that a weak cylindrical or toroidal lens (1,000 mm or greater) will introduce enough astigmatism to enable stigmatic microscopy but will not interfere significantly with the imaging performance of the system. This provides complementary advantages over other methods (PIV, OCT, Confocal) in that the temporal detail is only limited by the exposure time of a single image. With our particular microscope system, this worked for 500 ms exposures, but shorter exposures should be possible with a photo-multiplier tube or more sensitive camera. Moreover, because no scanning of a laser or light sheet is necessary, the rate of capture may ultimately be much faster.

The results shown for both ELISA and cell exclusion demonstrate that insights into the performance of ATPS driven assays can be captured using stigmatic microscopy. In ELISA, we found that viscous effects outweigh driving forces, while cell exclusion was found to be controlled by choosing either a critical or sub-critical ATPS condition. Moreover, we demonstrated immunoassay incubation times 16x faster than those previously demonstrated by our group.

Future work will further explore the application of stigmatic microscopy to multiplexed ELISA and will enable the derivation of complete flow fields of the droplet over time. We also believe additional work will enable accurate detection of the droplet shape and morphological changes as required by new applications.

In conclusion, we present a stigmatic microscope designed to enable facile measurement of convective mixing both inside and outside of ATPS droplets. The specific observations as well as general methodology should aid in the development of design principles for various ATPS applications. The ability to conveniently image 3D flows in the μm-mm regime is also envisioned to be useful in understanding the forces and dynamics that drive a number of microfluidic and droplet-based systems beyond ATPS systems.

## Author Contributions

CY, CO, TK, and ST: conceptualization. CY and CO: methodology. CO and CY: software. CY: validation. CO and CY: formal analysis. CY, CO, and TK: investigation. ST: resources. CY: data curation. CY, CO, TK, and ST: writing–original draft. CY, CO, TK, and ST: writing–review and editing. CO and CY: visualization. ST: supervision.

## Conflict of Interest Statement

The authors declare that the research was conducted in the absence of any commercial or financial relationships that could be construed as a potential conflict of interest. The handling editor declared a past collaboration with one of the authors ST.
