# Supplementary material for: Stigmatic Microscopy Enables Low-Cost, 3D, Microscale Particle Imaging Velocimetry in Rehydrating Aqueous Two-Phase Systems
Source: Front Chem. 2019 May 22;7:311. doi: 10.3389/fchem.2019.00311 (PMC6538919; doi:10.3389/fchem.2019.00311)
Supplement: Supplementary file 1 [file Table_1.DOCX]

Supplementary Materials for

**Stigmatic microscopy enables low-cost, 3D, microscale particle imaging velocimetry in rehydrating aqueous two-phase systems**

Cameron Yamanishi*^1,2^*^†^, C. Ryan Oliver*^2,3^*^†^, Taisuke Kojima*^1^*, Shuichi Takayama*^1,2,4^*

^1^ The Wallace H Coulter Department of Biomedical Engineering, Georgia Institute of Technology and Emory School of Medicine, Atlanta GA 30332 USA.

^2^ Department of Biomedical Engineering, University of Michigan, Ann Arbor, MI 48109, USA.

^3^ Department of Internal Medicine, University of Michigan, Ann Arbor, MI 48109, USA.

^4^ The Parker H Petit Institute for Bioengineering and Bioscience, Georgia Institute of Technology, Atlanta GA 30332 USA

* Corresponding author

† Equal contribution

Correspondence to: [takayama@gatech.edu](mailto:takayama@gatech.edu)

## Stigmatic microscope design


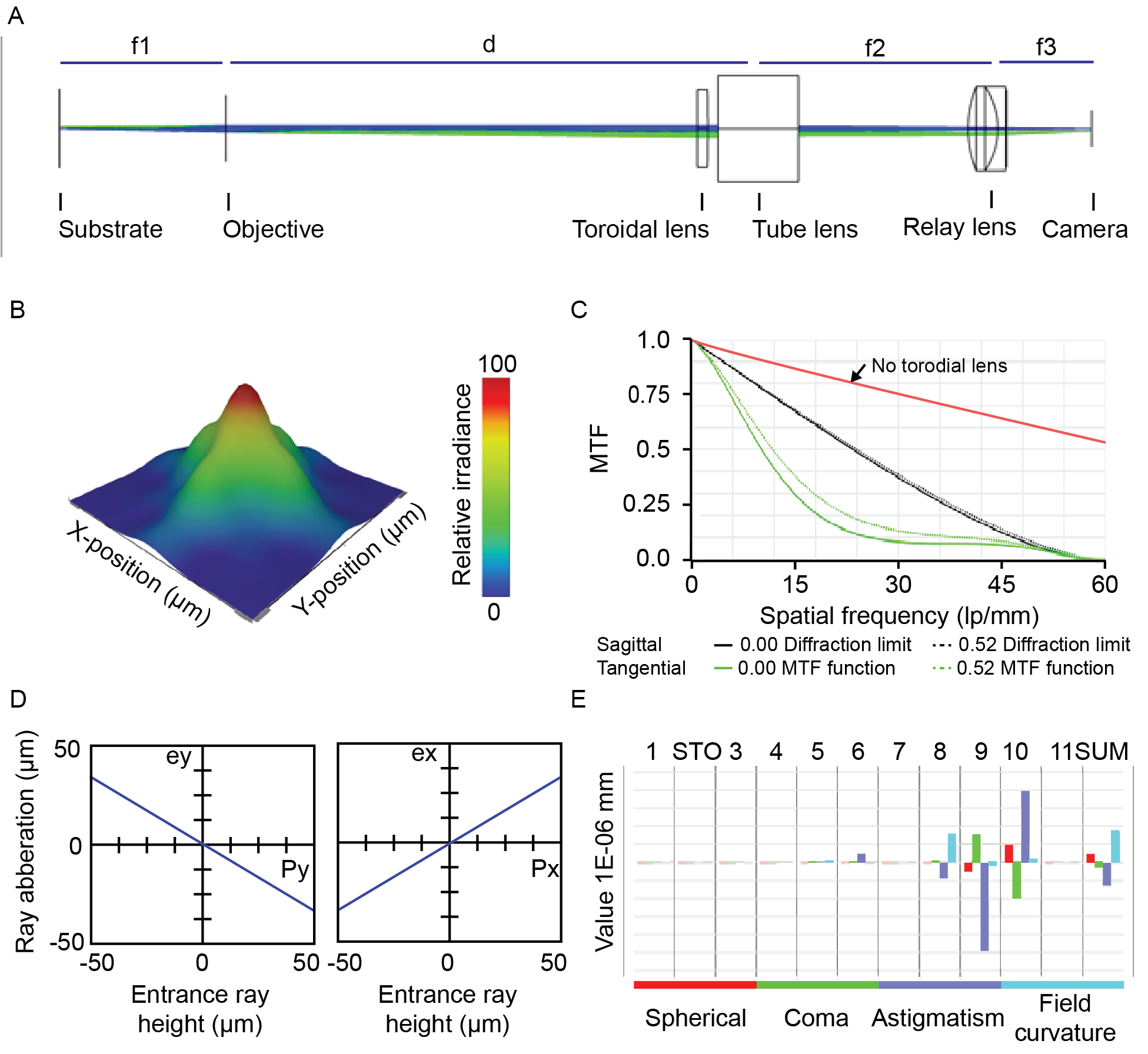


Figure S1. Model of stigmatic microscope. (A) Schematic. (B) Point-spread function (PSF). (C) Modulation transfer function with and without the toroidal lens. (D) Aberration ray fan plot showing astigmatism. (E) Aberration contribution from each surface in the microscope and a sum of the aberrations at the end.


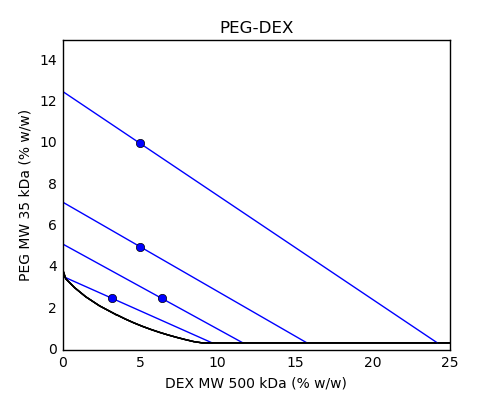


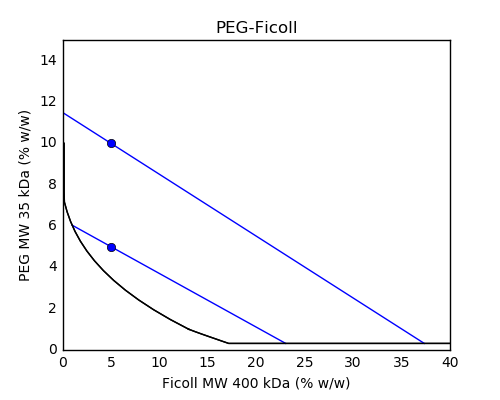


Figure S2. Phase diagrams for PEG-DEX and PEG-Ficoll systems. Circles indicate solutions used to fit tie-lines to the binodal curves based on measured volume ratios.


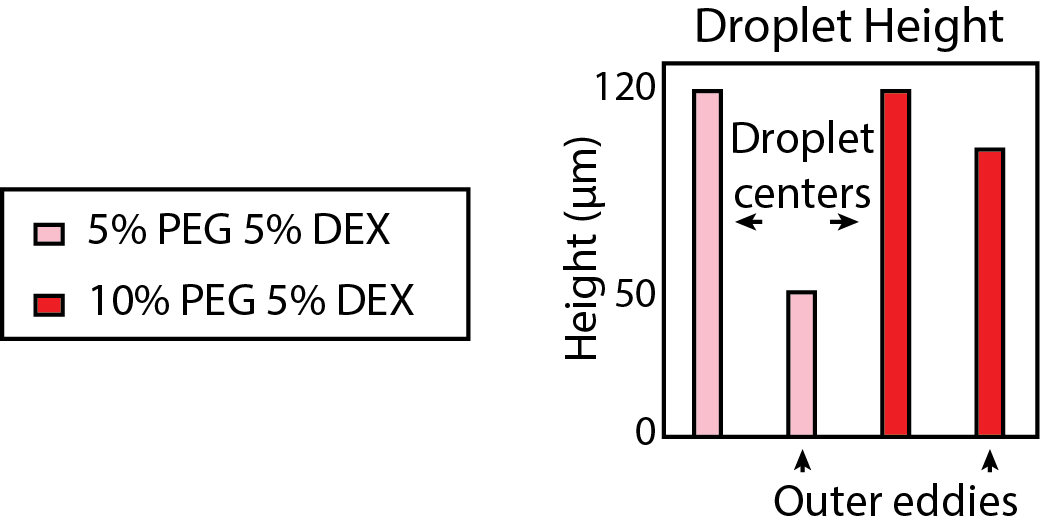


Figure S3. Vertical magnitude of flow traces within the 15-minute rehydration of 1 μL DEX droplets with 100 μL of PEG solutions.
